# Effects of *Kalimeris indica* on alcohol-induced liver injury through storing Nrf2/HO-1 pathway and gut microbiota

**DOI:** 10.3389/fphar.2024.1502096

**Published:** 2024-11-12

**Authors:** Mo-Fei Wang, Tong Sun, Shi-Yu Chen, Xue Wang, Hao Li, Jia-Qi Wang

**Affiliations:** ^1^ The Department of General Surgery, The Affiliated Hospital of Inner Mongolia University for the Nationalities, Tongliao, Inner Mongolia, China; ^2^ Shenyang Key Laboratory for Causes and Drug Discovery of Chronic Diesases, Liaoning University, Shenyang, China

**Keywords:** *K. indica*, liver injury, Nrf2, HO-1, gut microbiota

## Abstract

**Background:**

*Kalimeris indica (*L.*)* Sch. Bip., (*K. indica)* is a plant classified under the genus Kalimeris within the Asteraceae family. The herb of *K. indica* has been historically utilized as a traditional medicine. The consumption of excessive amounts of alcohol represents a lifestyle choice that can induce tissue damage and contribute to the development of various health conditions.

**Method:**

The HPLC-MS method was used to reveal the chemical composition of *K. indica* extract. HepG2 cells were used to test the *in vitro* oxidative stress. C57BL/6 mice were used to construct the *in vivo* alcohol-induced liver injury. H/E staining and serum ALT and AST levels were tested to assess the *in vivo* protective effect of ML (50 and 200 mg/kg). GSH, SOD, and CAT levels along with byproduct MDA levels were used to evaluate the *in vivo* oxidative stress. Immunohistochemical experiments were used to examine the *in vivo* Nrf2 and HO-1 levels. 16S rRNA gene-based profiling method was used to test the alteration in gut microbiota.

**Results:**

16 compounds were identified from *K. indica* extract. *K. indica* treatment reduced oxidative stress in HepG2 cells treated with 5% alcohol. H/E staining results showed that *K. indica* (50 and 200 mg/kg) alleviated liver injury caused by alcohol administration, eliciting a similar protective effect to the positive drug silymarin. Serum ALT and AST examination gave a consistent result, showing that ML could restore serum ALT and AST levels in mice treated with alcohol. Furthermore, *K. indica* could also restore GSH, SOD, CAT, and MDA levels in alcohol-treated mice, showing a potent effect on oxidative stress alleviation. Immunohistochemical experiments indicated that *K. indica* showed the liver protective effect through Nrf2/HO-1 pathway. 16S rRNA gene-based profiling revealed that alcohol treatment caused the alteration in gut microbiota, while *K. indica* treatment could result in a significantly richer variety of microbial communities compared to the alcohol group.

**Conclusion:**

*K. indica* (ML) has a protective effect on liver injury caused by alcohol administration. The Nrf2/HO-1 pathway and gut microbiota regulation were involved in the ML-induced liver protection. All the results indicate that *K. indica* has a potential in the treatment of alcohol-induced liver injury.

## 1 Introduction

*Kalimeris indica* (L.) Sch Bip (*K. indica)* is a plant belonging to the genus *Kalimeris* in the Asteraceae family. The entire herb of *K. indica* has been used as a traditional remedy, with the name Ma Lan (ML). As a common traditional medicine, it has a long history of medicinal use, with its first recorded application in the Bencao Shiyi (741 AD). The Chinese Pharmacopoeia states that ML can regulate vital energy flow, aid in digestion, and alleviate dampness and heat. Pharmacological studies have shown that it possesses anti-inflammatory and analgesic properties, as well as anti-tumor, anti-viral, and blood coagulation-improving effects. Our previous study showed that ML has a therapeutic effect on colitis-associated colorectal cancer (Wang et al., 2022).

The liver plays a crucial role in many important body functions. These include regulating blood volume (Li et al., 2021), supporting the immune system (Dąbrowska, et al., 2024), controlling growth signaling pathways, maintaining lipid and cholesterol balance (Heeren and Scheja, 2021), and breaking down certain compounds, including drugs (Almazroo, et al., 2017). The liver processes and metabolizes macronutrients to provide the energy needed for these functions. It also stores glucose in the form of glycogen and can produce glucose when needed. Additionally, the liver processes lipids, either using them for energy or storing them in other tissues. It also handles protein and amino acid metabolism, secreting many proteins into the blood and disposing of nitrogenous waste. Therefore, maintaining a normal liver function is very important.

How we live our lives, including what we eat, plays a key role in developing many diseases. Consuming excessive amounts of alcohol is a lifestyle choice that can cause damage to our tissues and contribute to the onset of several health conditions. Shockingly, harmful alcohol use leads to three million deaths around the world every year, which accounts for 5.3% of all deaths (Wu et al., 2023). Chronic and heavy alcohol consumption can lead to alcohol-associated liver disease (ALD), a major health issue that is among the leading causes of liver-related sickness and death. ALD encompasses a range of conditions from asymptomatic liver steatosis to fibrosis, cirrhosis, and alcohol-associated hepatitis (AH) and its complications. Among chronic heavy drinkers, around 8%–20% will develop alcohol-related cirrhosis. Of these patients, approximately 2% will advance to hepatocellular carcinoma (Seitz et al., 2018).

In this paper, we for the first time investigated the effect of ML on alcohol-induced liver injury and revealed the underlying mechanism, providing the scientific basis for the efficacy, material basis, and future scientific utilization of ML.

## 2 Materials and methods

### 2.1 Cells culture

The human hepatoma (HepG2) cell line was grown in Dulbecco’s Modified Eagle Medium (DMEM) with high glucose, supplemented with 10% fetal bovine serum (FBS), in a humidified atmosphere at 37°C with 5% CO_2_. The cells were allowed to grow until they reached approximately 80% confluence before the start of the experiments.

### 2.2 Preparation of K. indica extract

The preparation of ML was reported previously (Wang et al., 2022). Briefly, the dried herb of *K. indica* (2.5 kg) was soaked in water for 60 min and refluxed with water (10 L) for 3 times (1.5 h each). After combining the ML extraction, 0.43 kg of the extract was obtained under vacuum.

### 2.3 Chemical constituents identified from *K. indica* by LC-QTOF MS

Dried *K. indica* (10.0 g) was powdered and soaked in 100 mL 70% methanol solution (v/v) for 0.5 h and extracted by reflux condensation for 1 h. The mixture was then filtered, and the filter residue was treated with 50 mL of a 70% ethanol aqueous solution and refluxed for an additional 0.5 h. The two filtrates were combined and concentrated to a final volume of 15 mL. The extract of *K. indica* was subsequently placed in a 4°C environment for 24 h, after which the supernatant was obtained through filtration. The extraction was centrifuged at 3,000 rpm for 15 min and the supernatant was collected after filtering through 0.22 μm membrane for HPLC-QTOF-MS analysis.

Sample separation was carried on an Agilent 1260 HPLC system coupled with XBridge BEH C18 Column (5 μm, 4.6 mm × 250 mm) with a mobile phase consisting of water 0.1% formic acid (A) and acetonitrile containing 0.1% formic acid (B). The gradient elution program was set as follows: 0–5 min 3% (B), 5–35 min 3%–99% (B), 35–40 min 99%–10% (B). Agilent 6530 QTOF MS was used to achieve the identification, positive and negative mode was used in the analysis and the injection volume was 5 μL at the flow rate of 0.6 mL/min.

### 2.4 Measurement of intracellular ROS

Reactive oxygen species (ROS) levels were measured in HepG2 cells using a commercial kit protocol. Cells were pre-incubated with different concentrations of ML or without ML, and then exposed to 5% alcohol. After treatment, cells were washed and incubated with a ROS probe. Fluorescence intensity was measured using a microplate reader. Results were presented as fluorescent intensity, indicating ROS levels under different treatment conditions.

### 2.5 Animals and treatment

The study protocol received approval from the Ethics Committee for Animal Experiments of Liaoning University. All mice were housed in a controlled environment with regulated temperature and lighting. Following a one-week acclimation period, pathogen-free (SPF) grade male C57BL/6 mice were randomly divided into five groups (n = 7 for each group): Control (CON), Model (MOD), Low dose group (ML-L), High dose group (ML-H), and Silymarin (Sil). All mice were fasted for 2 h before daily dosing. Mice in the LD/HD/SD groups received ML or silymarin via gavage for 15 days. From the 10th days, 56% ethanol (5 g/kg bw) was administered to the mice, except for those in the control group, 2 h after ML/silymarin/saline administration. ML and silymarin were prepared using saline.

### 2.6 Measurement of liver parameters

The liver tissue samples, each weighing 60 mg, were mixed with 540 μL of saline in a cold environment to create a homogenate. The mixture was then centrifuged at 3,500 rpm and 4°C for 15 min to separate the components. The resulting supernatant was carefully collected for further analysis. Levels of reduced glutathione (GSH), catalase (CAT), superoxide dismutase (SOD), and malondialdehyde (MDA) were measured using commercially available kits (Shanghai Enzymelinked Biotechnology Co., Ltd., Shanghai, China). Additionally, the protein concentration was determined using a BCA reagent kit.

### 2.7 Measurement of serum parameters and histopathological analysis

The levels of serum ALT and AST, which are indicators of liver function, were measured using commercially available kits (Biosino Bio-Technology and Science Inc. Beijing, China). Fresh livers were carefully collected, and a small portion of each mouse liver was preserved in 4% phosphate-buffered paraformaldehyde for subsequent staining with a combination of hematoxylin and eosin (H&E). Following a 24-h fixation period, the livers were embedded in paraffin blocks and cut into thin 9-μm slices. These slices were then stained with H&E to visualize the cellular structure and were observed under a bright-field light microscope for further analysis.

### 2.8 Immunohistochemistry

To assess the expression of Nrf2 and HO-1 proteins, paraffin-embedded liver tissue samples were sectioned into 4 μm slices and then dewaxed and rehydrated. The sections were treated with a 10 μmol/L citrate buffer, microwaved for 15 min, cooled, and rinsed with PBS thrice. A peroxidase blocking solution (50 μL) was applied, followed by a 20-min incubation at room temperature, and another three rinses with PBS. The sections were then serum-blocked for 30 min. After removing the serum, diluted antibodies against HO-1 and Nrf2 (servicebio, China) were added and incubated overnight at 4°C. After three additional PBS rinses, the sections were incubated with horseradish peroxidase-labeled secondary antibodies for 50 min. DAB chromogenic solution was applied for staining, followed by reverse hematoxylin staining. The sections were dehydrated and sealed.

### 2.9 16S rRNA sequencing of colonic contents

Total genomic DNA samples were extracted using the OMEGA Soil DNA Kit (M5635-02) (Omega Bio-Tek, Norcross, GA, United States) following the manufacturer’s instructions, and were stored at −20°C before further analysis. PCR amplification of the bacterial 16S rRNA genes V3–V4 region was performed using the forward primer 338F (5′-ACT​CCT​ACG​GGA​GGC​AGC​A-3′) and the reverse primer 806R (5′-GGACTACHVGGGTWTCTAAT-3′). PCR amplicons were purified with Vazyme VAHTSTM DNA Clean Beads (Vazyme, Nanjing, China) and quantified using the Quant-iT PicoGreen dsDNA Assay Kit (Invitrogen, Carlsbad, CA, United States). After the individual quantification step, amplicons were pooled in equal amounts, and pair-end 2 × 250 bp sequencing was performed using the Illlumina NovaSeq platform with NovaSeq 6000 SP Reagent Kit (500 cycles) at Shanghai Personal Biotechnology Co., Ltd. (Shanghai, China). The data were analyzed on the online platform of Personalbio Genescloud (https://www.genescloud.cn/).

### 2.10 Statistical analysis

All data were presented as mean ± SEM. Differences between groups were compared using One-way ANOVA analysis. *p* value <0.05 was considered statistically significant.

## 3 Results

### 3.1 HPLC-Q-TOF-MS analysis of ML

**FIGURE 1:**
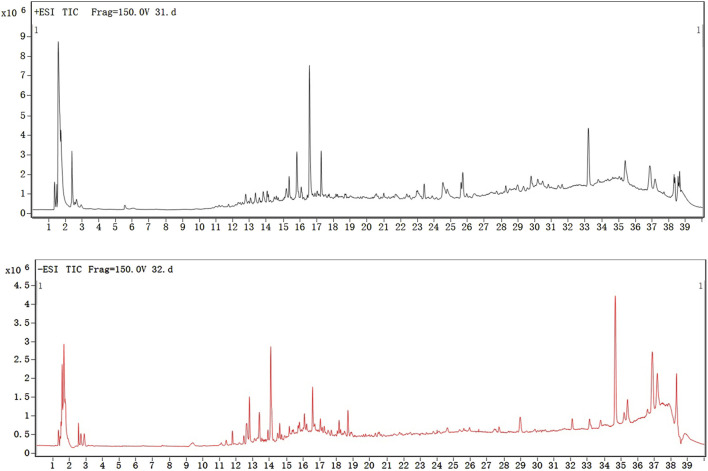
HPLC-MS results of ML.

We used HPLC-Q-TOF-MS experiment to reveal the components of ML. Consequently, 16 constituents were identified based on the high-resolution experimental ion peaks, including *p*-hydroxybenzoic acid, indole-3-carbaldehyde, chlorogenic acid, apigenin-7-*O*-glucoside, etc (Figure 1; Table 1).

**TABLE 1:** Results of HPLC-Q-TOF-MS.

t_R_/min	Compounds	Molecular formula	Experimental	Errors	Ion fragments
9.275	Protocatechuic acid	C_7_H_6_O_4_	153.0194	2.45	109.0296
9.417	Protocatechuate	C_7_H_6_O_4_	153.0191	1.28	109.0292, 108.0219
11.927	*p*-hydroxybenzoic acid	C_7_H_6_O_3_	137.0,244	0.42	112.8456
12.357	Indole-3-carbaldehyde	C_9_H_7_NO	146.0597	−0.92	131.0857, 107.0850
12.483	Chlorogenic acid	C_16_H_18_O_9_	355.1026	−2.97	164.0429, 353.0870, 191.0557
13.102	Ferulicacid	C_10_H_10_O_4_	239.0584	2	195.9059
13.277	Aesculetin	C_9_H_6_O_4_	177.0191	1.53	105.0342, 133.0285
13.344	Caffeic acid	C_9_H_8_O_4_	179.0348	3.1	153.0192, 143.8629, 110.0322
13.644	Apigenin-7-*O*-glucoside	C_21_H_20_O_10_	431.1924	2.01	268.4257
14.612	Rutin	C_27_H_31_O_16_	609.1457	−0.25	300.0342
14.853	Coumaric acid	C_9_H_8_O_3_	163.0395	3.17	145.8916, 134.0240, 119.0499
15.704	4,4-Dicaffeoylquinic acid	C_25_H_25_O_10_	515.1192	−0.88	179.0351, 191.0557, 353.0881
15.879	1-malonyl-3,5-dicaffeoylquinic acid	C_28_H_26_O_15_	601.1197	−0.03	191.0549, 179.0329
18.481	Quercetin	C_15_H_10_O_7_	301.0353	0.22	199.0383, 178.9988, 151.0025, 121.0350
25.657	Asperglaucide	C_27_H_28_N_2_O_4_	445.2136	−3.91	141.1132, 303.2643
35.383	Linoleic acid	C_18_H_32_O_2_	279.2325	0.1	261.2232, 276.5099

### 3.2 ML alleviates oxidative stress induced by alcohol in HepG2 cells

**FIGURE 2:**
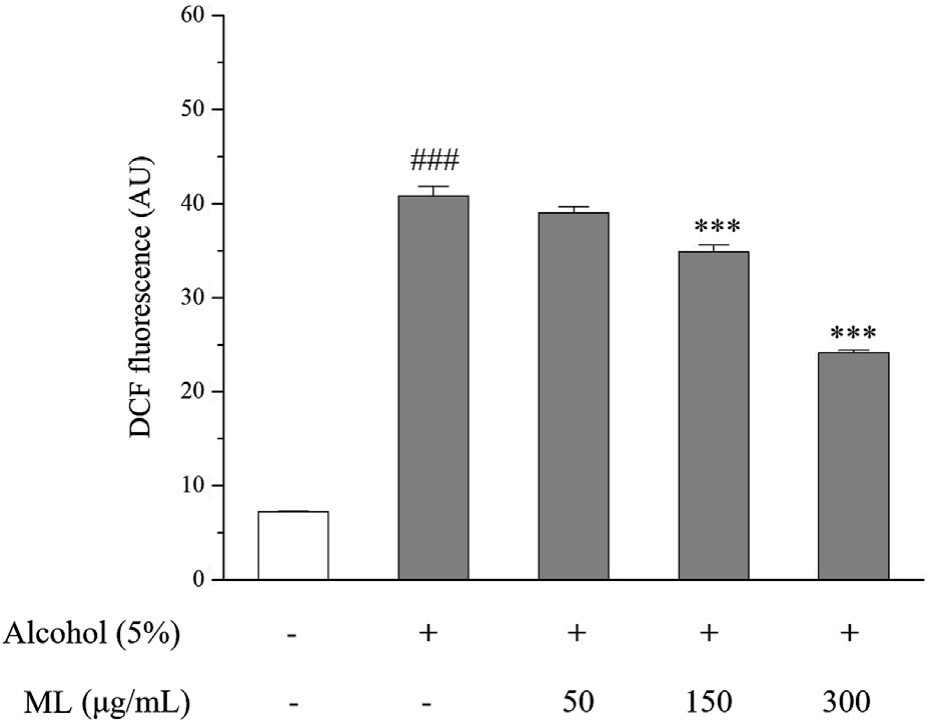
Oxidative stress degree indicated by DCFH-DA in HepG2 cells. Results were obtained from three independent experiments, expressed as Mean ± S.E.M. ^###^ *P <* 0.001 compared with the blank group; ***P < 0.001 compared with the alcohol group.

The fluorescence of DCF in each group was measured. As illustrated in Figure 5, a significant increase in absorbance was observed when treating 5% alcohol alone, indicating that alcohol stimulates the production of ROS in HepG2 cells. However, after incubation with moderate and high doses of ML (150 and 300 μg/mL), ROS levels were significantly reduced, as evidenced by the decreased absorbance. Therefore, ML was revealed to be capable of reducing alcohol-induced oxidative stress in HepG2 cells (Figure 2).

### 3.3 ML alleviates *in vivo* injury induced by alcohol in mice

**FIGURE 3:**
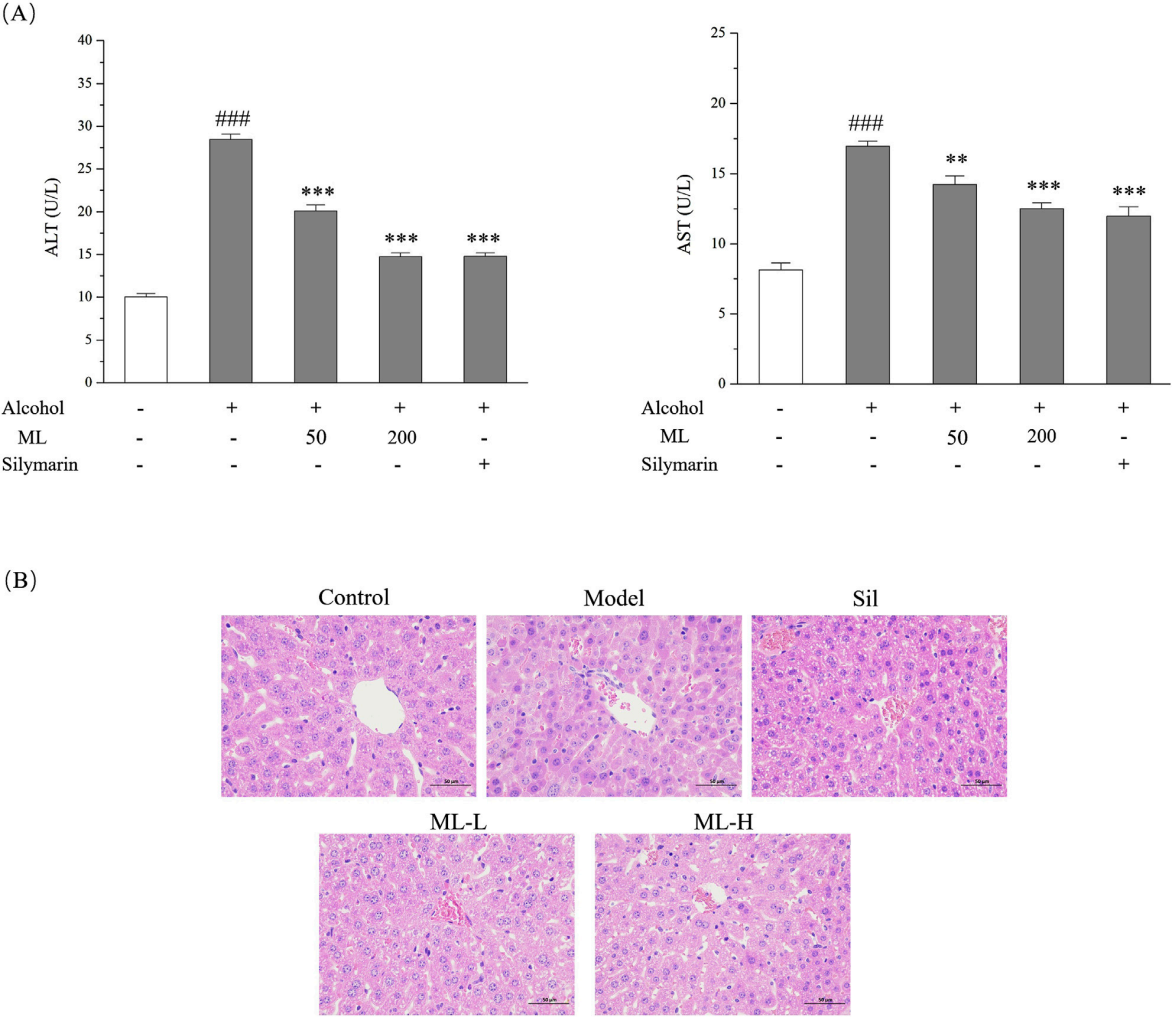
ML alleviates alcohol-induced liver injury (n = 7). **(A)** Serum ALT and AST levels of each group. Results were expressed as Mean ± S.E.M. ^###^ *P <* 0.001 compared with the control group; ^***^ *P <* 0.001 compared with the alcohol model group; ^**^ *P <* 0.005 compared with the alcohol model group. **(B)** Liver histopathological changes indicated by H&E staining experiments.

To assess the extent of *in vivo* liver damage caused by excessive alcohol consumption, we analyzed the levels of ALT and AST in the serum with silymarin as the positive drug since it had been reported to show a protective effect against alcohol-induced liver damage. As shown in Figure 3A, alcohol treatment significantly elevated ALT and AST levels in the serum, which were all ameliorated by the ML administration. H&E staining gave a similar result, showing the disordered structure of hepatic lobule, and plenteous large and small lipid vacuoles, which are typical pathological changes of ALD. The protective effect of the high-dose group was evident, showing a relatively normal structure of hepatic lobule and reduced lipid droplets compared with the model group. However, the protective effect of the low-dose group was relatively weak compared with the high-dose group (Figure 3B).

### 3.4 ML alleviates *in vivo* liver oxidative stress in mice

**FIGURE 4:**
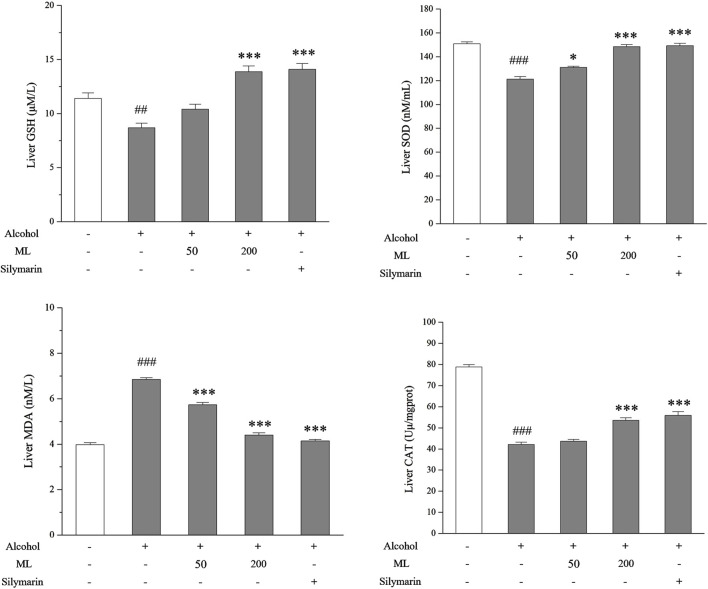
ML alleviates liver oxidative stress (n = 7). Liver nonenzymatic antioxidant GSH, SOD, and CAT levels along with byproduct MDA levels were tested as shown. ^##^ *P <* 0.005 compared with the control group; ^###^ *P <* 0.001 compared with the control group; ^***^ *P <* 0.001 compared with the alcohol model group; ^*^ *P <* 0.05 compared with the alcohol model group.

To assess the *in vivo* effect of ML on liver oxidative stress, the level of GSH, SOD, CAT, and MDA were tested. GSH is a non-enzymatic antioxidant that plays a vital role in eradicating free radicals, which helps to reduce oxidative stress. SOD and CAT are two enzymatic antioxidants. MDA is a byproduct of lipid peroxidation that can be inhibited by GSH, SOD, and CAT. As a result, upregulated GSH, SOD, and CAT levels and downregulated MDA levels were observed after alcohol treatment, while ML administration could partially reverse these changes in GSH, SOD, CAT, and MDA levels (Figure 4).

### 3.5 ML alleviates *in vivo* injury induced by alcohol in mice through Nrf2 and HO-2

**FIGURE 5:**
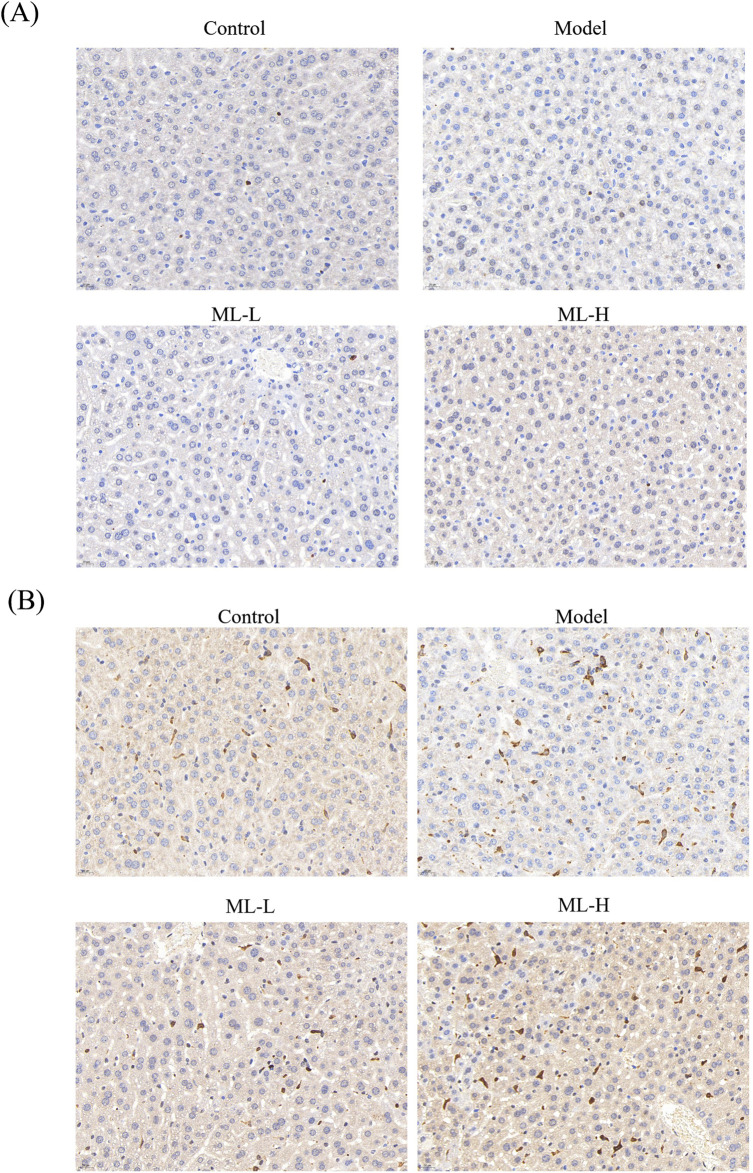
Immunohistochemical results of Nrf2 **(A)** and HO-1 **(B)**. Scarle bar: 20 μm.

To further study the mechanism of ML’s protective effect on alcohol-induced liver injury, immunohistochemical experiments were conducted. The results indicated that ML treatment further activated Nrf2 to elicit an anti-oxidative stress effect (Figure 5). Moreover, alcohol treatment suppressed the HO-1 level, while ML administration reversed the HO-1 level (Figure 5).

### 3.6 ML reverses gut microbiota in alcohol-treated mice

**FIGURE 6:**
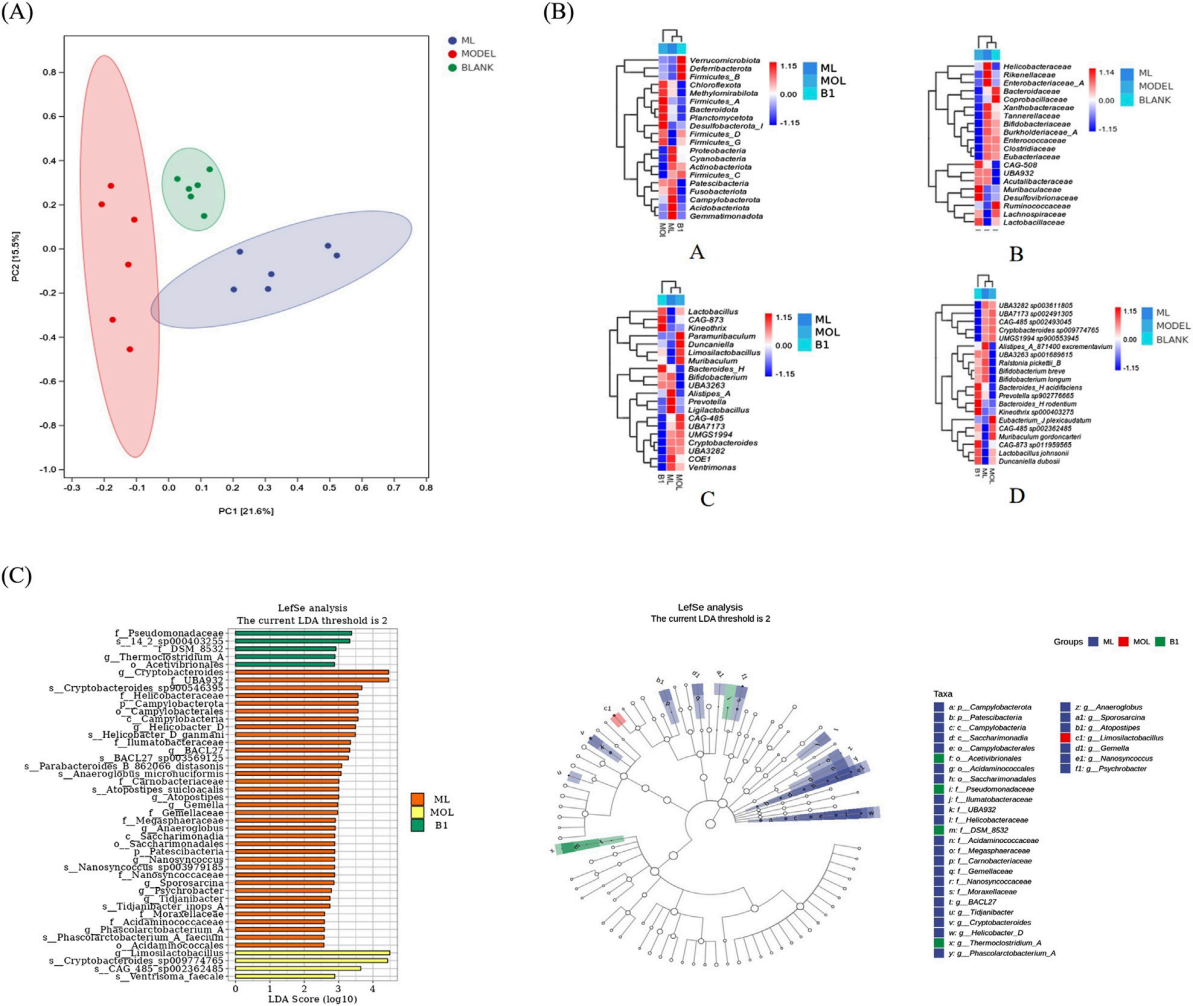
ML restores gut microbiota. **(A)** Results of PCoA analysis. **(B)** Heatmap of species composition of intestinal flora. **(C)** Results of LEfSe analysis.

To assess the effect of ML on microbiota, we conducted 16S rRNA sequencing experiments. The results showed that there is a certain degree of clustering between samples, indicating that the similarity of community composition within groups is relatively close, and there is obvious separation between groups without overlapping parts, suggesting significant differences between populations (Figure 6A). After intervention with ML, there were significant changes in the gut microbiota of mice. Detailed analysis indicated differences in microbial composition and abundance at the phylum, family, genus, and species levels among the blank group, model group, and ML treatment group. In addition to the differences in microbial communities in each group, it was suggested that ML can lead to different secretion of metabolites by microorganisms, thereby indirectly regulating liver metabolism through the “hepatointestinal axis” (Figure 6B). To be specific, the microbiota in the model group, which is significantly different from the blank group and the ML group, is mainly *Limosilactobacillus*. The ML group has a significantly richer variety of microbial communities compared to the other two groups, with the representative species being the *Cryptobacteroides* genus, UBA932 family; The genus *Helicobacter* in the family Helicobacteraceae (Figure 6C).

## 4 Discussion

Hepatic steatosis/AFL, which is a common feature at the beginning of ALD, is characterized by increased NADH concentration during alcohol metabolism, leading to impaired fatty acid oxidation and tricarboxylic acid cycle activity. The Nrf2 pathway has been implicated in the development of ALD, with oxidative stress triggering changes in the Keap1-Nrf2 interaction. Research has shown that Keap1 can be rapidly degraded in response to acute stress in mice. However, *in vitro* studies suggest that Keap1 may be regenerated in the presence of ongoing oxidative stress, allowing Nrf2 to evade degradation. Additionally, upregulated p62 has been found to competitively bind to the Keap1 interaction region, leading to the degradation of misfolded proteins and protein aggregates induced by oxidative stress (Bae et al., 2013). Finally, activation of Nrf2 can result in the expression of antioxidant and detoxification genes, such as heme oxygenase-1 (HO-1). Research has shown that certain medications can act as protective agents against ALD by triggering the Nrf2 pathway (Sun et al., 2018; Zhao et al., 2018). On the other hand, mice deficient in Nrf2 and given ethanol had a higher mortality rate compared to normal mice (Bataille and Manautou, 2012), suggesting that targeting the Nrf2/HO-1 signaling axis could be an effective approach in the treatment of ALD (Li et al., 2020). In this paper, we found MLC has the potential to activate Nrf2/HO-1 pathway, promoting an enhanced antioxidant pathway, which is considered to be one of its mechanisms exerting a protective effect on ALD.

Moreover, according to the traditional Chinese theory, the liver can store the blood. Generally, the relationship between the liver and blood can be illustrated as the following: 1. The liver stores most of the blood in the human body. When the body is active, blood flows to other organs and tissues to support their normal activity. During rest or decreased physical activity, most of the blood flows back to the liver. 2. The liver can regulate the amount of blood flowing in and out. When the body is active, other organs require more blood, so the amount of blood flowing out from the liver increases. When the body is relatively still, the demand for blood in various parts of the body decreases, and the amount of blood flowing back to the liver increases. 3. The liver stores blood after the meridians, reducing the risk of bleeding caused by excessive blood flow. If the liver function is abnormal, it may result in symptoms such as blood deficiency, excessive dreaming, and anxiety due to inadequate blood storage in the liver. The traditional Chinese theory also recorded that ML can cool blood and stop bleeding, indicating that ML has a certain effect on liver and blood. Although we demonstrated its liver protective effect in this paper, its effect on liver-related blood circulation should be investigated in future papers.

## Data Availability

The datapresented in the study are deposited in the Zenodo repository, accession number: 10.5281/zenodo.13841388.
